# A simple flow-injection method for the determination of blood glucose using a Technicon immobilized enzyme coil

**DOI:** 10.1155/S1463924689000192

**Published:** 1989

**Authors:** Sveinbjörn Gizurarson

**Affiliations:** Novo Symbion Drug Delivery Group Haraldsgade 68 Copenhagen Ø DK-2100 Denmark

## Abstract

The applicability of a single-channel flow-injection system with immobilized enzyme coil (Technicon) and UV detection to the determination of glucose is described. The method was used for a pure glucose solution and for serum. The detection limit was 0.10 mM, the rate of determination was 20-40 per hour and the precision was satisfactory. The system is very simple and practical when many analysis are to be determined periodically.


Journal of Automatic Chemistry Vol. 11, No. 2 (March–April 1989), pp. 87–88

### A simple flow-injection method for the determination of blood glucose using a Technicon immobilized enzyme coil

**Sveinbjörn Gizurarson**

*Novo Symbion Drug Delivery Group, Haraldsgade 68, DK-2100 Copenhagen Ø, Denmark**

*The applicability of a single-channel flow-injection system with immobilized enzyme coil (Technicon) and UV detection to the determination of glucose is described. The method was used for a pure glucose solution and for serum. The detection limit was 0·10 mM, the rate of determination was 20–40 per hour and the precision was satisfactory. The system is very simple and practical when many analysis are to be determined periodically.*

### Introduction

The determination of glucose in serum, plasma and other biological samples is frequently required in clinical chemistry. In recent years various methods for the determination of glucose have been based on flow-injection analysis (FIA) with enzyme columns that are not commercially available [1]. This paper describes a simple determination of glucose by FIA in combination with a Technicon immobilized enzyme coil for the Auto-Analyzer II system [2,3].

### Experimental

*Reagents*

All reagents were of analytical-reagent grade and distilled water was used throughout. The immobilized enzyme-containing coil is commercially available from Technicon Instruments (Krondalvej 8, Dk-2610, Rødovre, Denmark) (Technicon Product no. T10-0003) ready for use. The carrier solution contained 55·46 mM PIPES buffer [piperazine-*N*,*N*′-bis(2-ethane)-sulphonic acid], 2·63 mM magnesium chloride, 0·015% Brij-35 (polyoxyethylene 23-lauryl ether), 0·80 mM ATP (adenosine 5′-triphosphate) isolated from equine muscle and 1·10 mM NAD (β-nicotinamide adenine dinucleotide) from yeast, all commercially available from Sigma Chemical Company (P.O. Box 14508, St. Louis, MO 63178, USA). Finally, the solution contained 2·79 mM MgEDTA (ethylenediaminetetraacetic acid magnesium salt) (Fluka, CH-9470 Buchs, Switzerland).

Glucose standards were prepared by diluting D(+)-glucose (Sigma) in saturated benzoic acid solution with water. Standard serum (K-86, bovine and horse serum for *in vitro* diagnostics; Nycomid Skandinavia, P.O. Box 4284, Torshov N-0401 Oslo 4, Norway) containing 6·03–6·49 mM glucose was used.

* This work was carried out at the Royal Danish School of Pharmacy, Department of Pharmaceutics, Universitetsparken 2, DK-2100 Copenhagen Ø, Denmark.

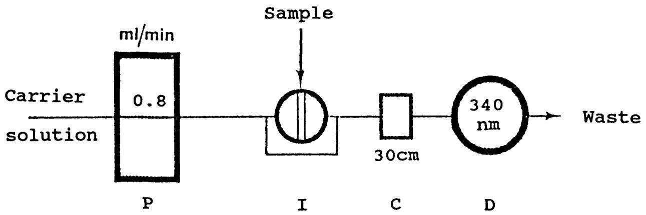

*Figure 1. Manifold for the flow injection analysis of glucose in biological fluids. The manifold consists of a peristaltic pump (P), injector (I), enzyme coil (C) and UV detector (D).*

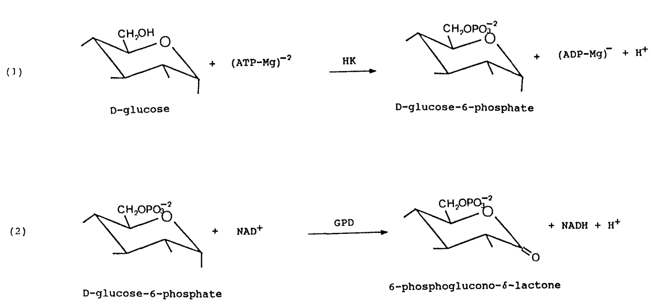

*Figure 2. Schematic illustration of the enzymatic reactions in the Technicon immobilized enzyme coil. The coil consists of two types of enzymes, hexokinase (E.C. 2.7.1.1) from yeast and glucose-6-phosphate dehydrogenase (E.C. 1.1.1.49) from* Leuconostoc mesenteroides *in co-immobilized form, coated on the inner wall of a disposable 30-cm nylon tube. Hexokinase (HK) catalyses the phosphorylation of glucose by ATP (1), then dehydrogenation by glucose-6-phosphate dehydrogenase (GPH) follows with reduction of $NAD^+$ to the coloured NADH (2).*

*Apparatus*

The manifold was build as shown in figure 1. An SHS 200 peristaltic pump (FIAtron Systems, 510 S. Worthington Street, Oconomowog WI 53066, USA), an actuator provided by Bifok (Box 124, Malmvägen 28, S-19122, Sollentuna, Sweden) and an Ultrospec 4050 UV spectrophotometer (Pharmacia LKB Biotechnology, Herredsvejen 2, DK-3400 Hillerød, Denmark), fixed at a wavelength of 340 nm and coupled with an RE 511 Kompensationschreiber (Bruwn Boviri, Norm Elec. Horsvinget 7, DK-2630, Taastrup, Denmark) recorder were used. Polyethylene tubing of 0·50 mm i.d. was used throughout the system.

*Procedure*

The sample (22 μl) was injected manually into the carrier line with a syringe. The flow-rate was 0·52 ml $min^{-1}$ and

0142–0453/89 $3.00 © 1989 Taylor & Francis Ltd.

experiments were performed at room temperature. After injection, the mixture was led through the enzyme coil cell where the enzymatic reactions took place (figure 2), through a flow cell and from there to waste. The retention time was about 3 min.

The sample concentration was calculated on the basis of the peak height, by reference to a calibration graph obtained by linear regression.

### Results and discussion

Calibration data for glucose standards were measured at concentrations 0·00, 0·56, 1·11, 1·67, 2·78 and 3·33 mM with six determinations at each level. The correlation coefficient was 0·994 and the mean coefficient of variation (CV) was 1·5%, ranging from 0·0 to 3·6%. Carry-over was tested as described by Andersen and Hannibal [3] and was found to be 2%. The detection limit was found to be 0·10 mM, which covers the normal range of plasma glucose (4·2–6·7 mM) and hypo and hyperglycaemic levels resulting from metabolic disorders. The mean glucose concentration from a serum standard was found to be 6·3 mM (CV 2·75%, $n$ = 6), compared with the declared concentration of 6·03–6·49 mM. When the glucose level was determined in serum or plasma, no interference of proteins was observed. The rate of determinations was 20 per hour because of the high viscosity, but dilution (3 : 5) with 70% ethanol increased the capacity to 40 determinations per hour. When full blood samples are analysed, addition of heparin (15 µl for a ca 200-µl sample) is needed, followed by centrifugation.

In conclusion, the system is simple and practical when many analyses are to be performed periodically.

### Acknowledgements

This work was supported by NOVO Industry A/S, Bagsvaerd, Denmark. The author thanks Mrs Lene Lykke Rasmussen for skilful technical assistance. Ib Andersen, Department of Clinical Chemistry, Copenhagen Country Hospital, Herlev, Denmark, is thanked for fruitful discussions and Arne Jensen, Department of Pharmaceutical Chemistry, Royal Danish School of Pharmacy, is thanked for providing the pump and all tubing.

### References

1. Hansen, E. H., in *Flow Injection Analysis* (Polyteknisk Forlag, Lyngby, Denmark, 1986), p. 66.
2. *Multichannel Biochemical Analyzers*, Technicon Method No. SF4-0046FA8 (Technicon Instruments, Tarrytown, NY, 1978).
3. Anderson, I. and Hannibal, S., *Journal of Automatic Chemistry*, **5** (1983), 188.

### Short courses

Loughborough University of Technology, UK, has announced the following short courses for 1989:

Fluorescence and Luminescence Spectrometry – 26–30 June 1989. Fee £480 including residence and all meals (£450 if paid with booking form). Non-residents £405 (£375).

Statistics for Analytical Chemistry – 11–14 July 1989. Fee £385 including residence and all meals (£355 if paid with booking form). Non-residents £325 (£295).

Flow Injection Analysis – 12–14 July 1989. Fee £325 including residence and all meals (£300 if paid with booking form). Non-residents £275 (£250).

*For further details please contact: Mrs J. E. Stirling, Department of Chemistry, Loughborough University of Technology, Loughborough, Leics. LE11 3TU. Telephone: (0509) 222549.*

HPLC Technology and Applied Chromatography Systems are holding five HPLC Beginners Training Courses during 1989. Each course will last three days and will include both practical and discussion sessions. The courses are held at The Deanwater Hotel, Woodford, Cheshire. All purchasers of HPLC Systems from ACS receive a complimentary place on the course.

*For further information please contact: Applied Chromatography Systems, The Arsenal, Heapy Street, Macclesfield, Cheshire SK11 7JB. Telephone: (0625) 34575.*

Chemserve have announced two courses for 1989:

Fluorine Spectroscopy Workshop – 10–11 April 1989.

Mass Spectrometry for Beginners – 17–18 April 1989.

*For further information, please contact: Chemserve, UMIST, PO Box 88, Manchester M60 1QD. Telephone: 061 228 7700.*

The University of Liverpool is holding a short course on Modern Spectroscopic Techniques, 2–7 April 1989.

*For further information, please contact: Dr A. Hodgson, Dept. of Chemistry, University of Liverpool, PO Box 147, Liverpool L69 3BX.*

Royal Society of Chemistry Residential School – 28–31 March 1989. Computer Methods in UV, vis and ir Spectroscopy, Polytechnic of Wales.

*For further information, contact Ms L. Hart, RSC, 30 Russell Square, London WC1B 5DT. Telephone: 01-631-1355.*

